# Health related quality of life (HRQOL) from the perspective of patients with chronic whiplash-associated disorders (WAD) in Sweden

**DOI:** 10.1186/s12891-025-08397-2

**Published:** 2025-02-14

**Authors:** Koustuv Dalal, Gunnel Peterson, Anneli Peolsson

**Affiliations:** 1 Division of Public Health Science, Department of Health Sciences, Mid Sweden University, Sundsvall, Sweden; 2 Centre for Clinical Research Sörmland, Uppsala University, Eskilstuna, Sweden; 3 Department of Health, Medicine and Caring Sciences, Physiotherapy, Linköping University, Linköping, Sweden; 4 Occupational and Environmental Medicine Centre and Department of Health, Medicine and Caring Sciences, Unit of Clinical Medicine, Linköping University, Linköping, Sweden

**Keywords:** Health related quality of life, EQ-5D, EQ-5D VAS, WAD, Sweden

## Abstract

**Background:**

The current study investigated Whiplash Associated Disorders (WAD) and health related quality of life (HRQOL) from the perspective of Swedish patients. Another aim was to assess medicine consumption and income loss due to WAD.

**Method:**

The present study was a planned secondary analysis using baseline data from a prospective, multicentre randomized controlled trial. The study participants were WAD patients, victims of four-wheel motor vehicle collisions at least six months but not more than five years ago. Neck Disability Index and HRQOL were measured. HRQOL was measured by the EQ-5D instrument. Cross tabulations, Box Plots, and regression analyses were performed.

**Trial registration section:**

The study was registered before data collection started (ClinicalTrials.gov Protocol ID: NCT03022812, initial release 12/20/2016).

**Results:**

There were 137 WAD participants (78.8% women), and almost three-fourths (74.5%) were married. The majority (54.7%) of the WAD patients were in white-collar jobs, followed by blue-collar jobs (35%) and students /unemployed (10.2%). Both consumption of medicine for neck pain and income loss due to WAD have a negative relation with the Neck Disability Index (NDI). On average, EQ-VAS for WAD women is 58.21 (± 17.625), and for men, it is 61.11 (± 16.444). WAD patients with a university education have the highest EQ-VAS average of 60.42 (± 17.738).

**Conclusions:**

The low HRQOL seen in WAD patients in this study should warrant the attention of the medical fraternity, researchers and policymakers.

## Introduction

Whiplash-associated disorders (WAD) are emerging health problems drawing the interests of researchers as well as medical practitioners. WAD easily transit to chronicity; approximately half of those after a whiplash injury develop chronic disability and pain [28, 32, 35]. WAD are often the results of rear-end motor vehicle crashes where the victim’s head experienced injuries by sudden acceleration-deceleration due to quick changes in the kinetic and static energies of the body and the vehicle [3–5, 10, 13, 17]. WAD is also frequently observed in sports, especially body contact games like ice hockey. Sports account for approximately 10% of WAD [2]. WAD causes several physical and mental health problems, including neck pain and stiffness, iterative pain, blurred vision, headaches, dizziness, constant tiredness, different physical dysfunctions, stress, anxiety, Post Traumatic Stress Disorder (PTSD) and other emotional and cognitive disorders [5, 14, 17, 24, 35]. WAD victims often have the risk of persistent impaired neck mobility, which grossly affects their daily activities and, hence, their quality of life [23], [18].

Quality of life (QOL) emerged as an important concept and research target for the medical and health sciences as it provides necessary information for patients’ care, rehabilitation and better societal participation. The World Health Organization’s (WHO) definition of QOL is: “An individual’s perception of their position in the life in the context of the culture in which they live and in relation to their goals, expectations, standards and concerns” [38]. Self-reported QOL helps policymakers provide improved treatment, care, and rehabilitation. It is also used for medical decision-making, including cost-effectiveness analysis. Patient-reported outcome measures (PROMs) of QOL are gaining in-depth interest as helpful instruments for decision-makers and researchers. It provides a comprehensive ‘value’ of the healthcare services and systems as the patients can express their own utility preferences. EQ-5D from the EuroQOL group is one of the most widely used PROM instruments worldwide for assessing health related quality of life (HRQOL) [16, 40]. The EQ-5D instrument consists of two parts: utility index (questionnaire containing five dimensions with three or five alternatives) and visual analogue scale (EQ-VAS). The five-dimensional questionnaires provide the societal value of HRQOL [25]. EQ-VAS derives patients’ self-reported utility, as a global assessment of health which reflects their health status [9].

EQ-VAS reporting is very common for HRQOL research because of its high response rate and completion level [6, 7, 9, 40]. EQ-VAS is an essential instrument in Sweden for effectively capturing patients’ important aspects and issues, as the Swedish people highly value their health status [1].

Several studies have examined the factors related to WAD and the connection between whiplash injuries and HRQOL [14, 26, 35, 37]. Sweden has an estimated WAD incidence of 3 out of 1000 inhabitants per year [2]. Therefore, there is an urgent need to understand the HRQOL of WAD patients in Sweden. The current study investigated chronic WAD and HRQOL from the perspective of Swedish patients who have not previously performed. Another aim was to assess medicine consumption and income loss due to WAD among a group of Swedish patients.

## Method

The present study was a planned cross-sectional secondary analysis using baseline data from a prospective, multicentre randomized controlled trial (RCT). Participants were constituted from ten Swedish county councils, and the study was conducted during April 6, 2018, and September 15, 2020. Written informed consent was obtained from all participants before allocation. There were 140 patients at the beginning, but three WAD patients did not reply to our target variables, resulting in 137 WAD participants for the current study.

The study participants were whiplash injury patients, victims of a four-wheeled motor vehicle accident at least six months but not more than five years ago. Initially, study information was advertised in print, social media, and university websites, inviting participants to participate. Interested participants (18–63 years) answered a short online survey. The survey contents were about whiplash injury-related neck symptoms and the time of the injury occurrence. WAD patients with an average neck pain intensity over the last seven days > 20 mm based on a 100-mm Visual Analog Scale (VAS) and > 20% based on the Neck Disability Index (NDI percentage score, 0–100%) were the target population.

Then, a telephone interview was performed to confirm their capability to join the study based on the inclusion and exclusion criteria. To be included, the WAD patients should be able to participate in the exercise interventions and have symptoms of neck pain, stiffness, or cervical radiculopathy within the first week after the injury. They must have regular access to a smartphone, tablet, or computer with an internet connection.

Exclusion criteria were: head injury and losing consciousness during the accident; amnesia prior to or after the accident; change in mental status; focal neurological alteration in olfactory and gustatory mechanism; previous episode of severe problem in the cervical spine. Patients diagnosed with other serious physical health problems, tumours, infections or malignancies in the spine were excluded. Patients with severe neck problems who were absent from their jobs for more than 30 days prior to the current WAD episode, having surgery in the cervical spine, or other prevailing physiological pain were excluded from the participant’s list. Patients having any other diseases, injuries or mental health problems which could deter them from full participation in the study or having alcohol or drug abuse problems were excluded. Also, participants who were unable to understand and write in Swedish were excluded. Participation in the earlier NSE study was excluded from the current study [18]. More details of the study procedure and methods are available elsewhere [21].

Lastly, before recruitment, an experienced physiotherapist physically examined the patients’ necks. The examination was based on neck pain and clinical musculoskeletal signs (WAD grade II) or WAD grade III (grade II and neurological signs) [31].

Demographic variables were reported at baseline and included age in years, sex, education level, marital status, employment sector, pain medication and income lost due to the whiplash injury.

EQ-5D-3 L Index and EQ-5D VAS (0–100 scale, where 0 denotes the worst imaginable health state and 100 refers to the best imaginable health state) were used for measuring HRQOL [30]. Minimal Clinically Important Differences (MCID) for EQ-5D for musculoskeletal patients were between 0.03 and 0.54 [15].

NDI was used, which is the most widely used, reliable, and valid instrument for determining neck pain [21]. NDI has ten items of neck-related disability (0% denotes the absence of disability, and 100% denotes major disability). A systematic review suggested that short-term therapy goals should aim for a minimum change of 5 points on the NDI for patients with WAD grades I and II and up to 10 points for those with higher severity [19]. NDI and EQ values are normally distributed [22].

### Statistical analysis

Cross tabulations were used to visualize the percentage distribution of the variables of interest (consumed medicine and lost income due to WAD and EQ-5D) with the demographic variables. Box plots demonstrated the EQ-VAS distribution among the demographic variables. The five dimensions and their three alternatives were also used against each demographic category to understand the actual percentage better. Ordinary least squares (OLS) regression models were used to define the societal values of EQ-5D-3 L [1]. For HRQOL presentation, including VAS simple percentage, average, standard deviation and median indicate expected values and provide necessary information [7, 8].

We assessed the impact of the NDI effect on the HRQOL of the WAD patients using the visual analogue scale (EQ-VAS) of HRQOL. A linear regression line was drawn.

Analyses were conducted in IBM SPSS V28. The significance level was tested at *p* < 0.05.

## Results

A total of 137 participants (mean age 40.5 years (SD ± 11.5), 79% women, mean duration of WAD 26 months (SD ± 16.8)) were included in the study. Almost three-fourths (74.5%) were married. The majority (54.7%) were in white-collar jobs, followed by blue-collar jobs (35%) and students or unemployed (10.2%). The average EQ-VAS was 58.79 (± SD 17.41), the EQ-5D societal score for WAD patients was 0.60 (± SD 0.25).

Table 1 shows that participants aged between 30 and 49 consumed proportionally less pain medicine (78.4%) for their neck pain than their peers aged 50 years and above (90.3%), while the income loss due to WAD has the opposite proportional direction, i.e. 55.4% vs. 45. 3%, respectively. Women proportionally consumed more pain medicine (86.9% vs. 69%) and lowered their income due to WAD (55.1% vs. 44.8%) more than male participants.

**Table 1:** Demographic variables and participants’ income loss and medicine consumption due to whiplash-associated disorders

	*N*	Consume medicine *n* (% of *N*)	Lost income from WAD *n* (% of *N*)
**Age**			
Up to 29	31	27 (87.1%)	17 (54.8%)
30–49	74	58 (78.4%)	41 (55.4%)
50 and above	31	28 (90.3%)	14 (45.3%)
**Sex**			
Female	107	93 (86.9%)	59 (55.1%)
Male	29	20 (69%)	13 (44.8%)
**Education**			
High school	68	57 (83.8%)	38 (55.9%)
University	59	50 (84.7%)	29 (49.2%)
Others	9	6 (66.7%)	5 (55.6%)
**Marital status**			
Single	26	20 (76.9%)	12 (44.4%)
Married/ live with partner	102	87 (85.3%)	57 (56.4%)
Other	8	6 (75%)	3 (37.5%)
**Employment sector**			
White collar job	74	63 (85.1%)	36 (48.6%)
Blue collar job	48	39 (81.3%)	28 (58.3%)
Student or unemployed	14	11 (78.6%)	8 (57.1%)

WAD participants who are married or living with partners consume a very high proportion (85.3%) of pain medicine and a high proportion (56.4%) of them lowered their income.

WAD participants working as white-collar employees consume the highest proportion (85.1%) of pain medicine. However, blue-collar workers have the highest proportion (58.3%) of income loss.

Table 2 shows the binary logistic regression results. Both consumption of pain medicine for neck pain and income loss due to WAD have a negative relation with the Neck Disability Index (NDI). Men are less likely (OR 0.332, CI. 0.111–0.994) to consume pain medicine for their WAD compared to women.

**Table 2:** Logistic regressions examining the association between the Neck Disability Index (NDI) and demographic factors against medicine consumption and income loss in patients with whiplash-associated disorders

	Consume medicine			Income loss from WAD		
	aOR	95% Conf. Interval		aOR	95% Conf. Interval	
**NDI**	0.934	0.889	0.981**	0.874	0.832	0.919***
**Age**						
Up to 29	Ref.			Ref.		
30–49	5.447	0.892	33.277	1.103	0.261	4.665
50 and above	2.380	0.285	19.860	2.226	0.468	10.581
**Gender**						
Male	0.332	0.111	0.994**	0.758	0.266	2.161
Female	Ref.			Ref.		
**Education**						
High school	Ref.			Ref.		
University	0.672			0.729	0.282	1.884
Others	2.275	0.348	14.881	1.154	0.181	7.379
**Marital status**						
Single	Ref.			Ref.		
Married/ live with partner	0.439	0.122	1.582	0.505	0.163	1.562
Other	0.685	0.065	7.260	1.162	0.118	11.473
**Employment sector**						
White collar job	Ref.			Ref.		
Blue collar job	1.664	0.522	5.306	0.749	0.294	1.906
Student or unemployed	5.004	0.663	37.763	0.950	0.153	5.888

*aOR* adjusted Odds Ratio

**p < 0.05

***p < 0.001

### Quality of life

**Fig. 1:**
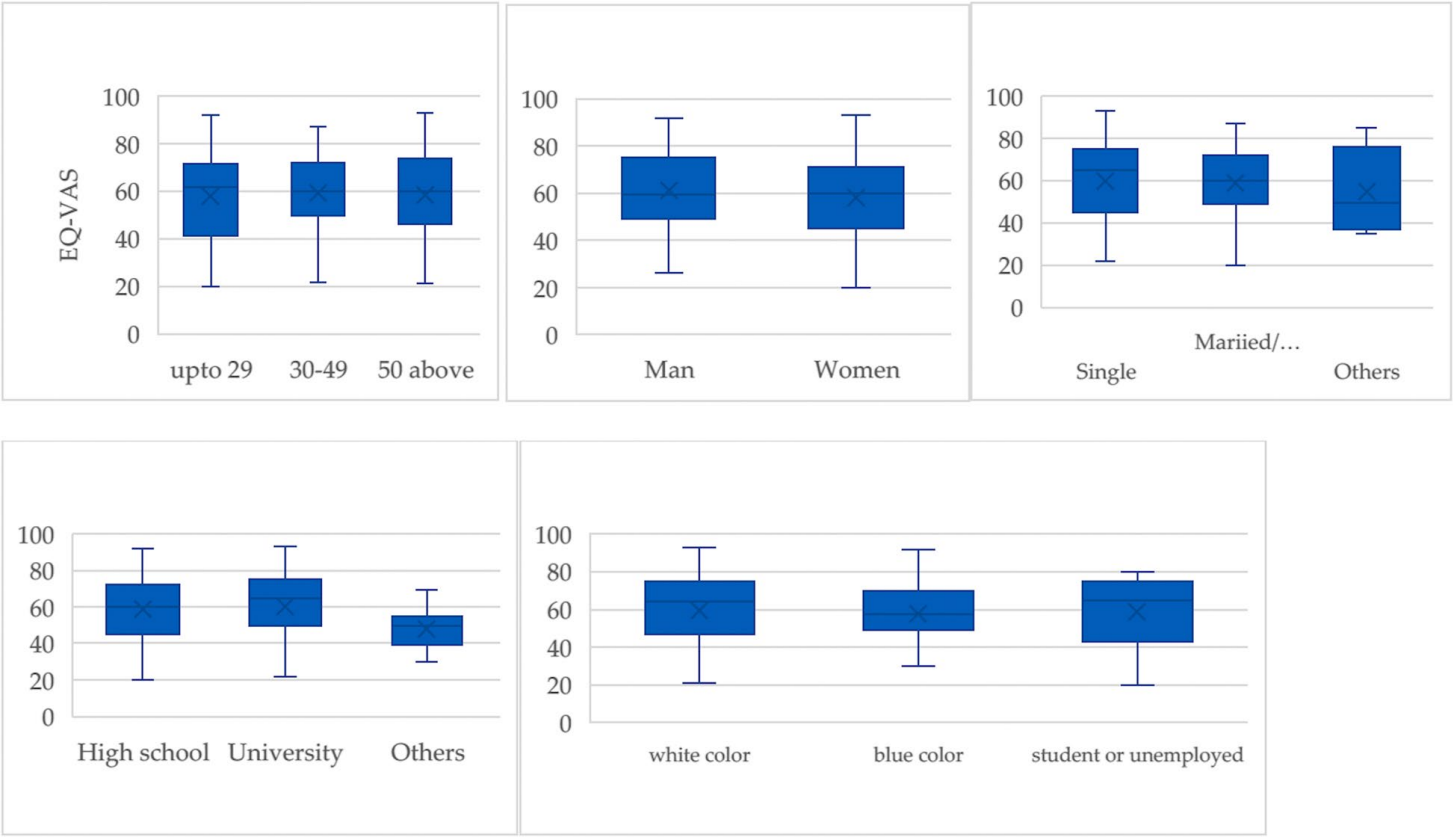
Box-plots showing EQ-VAS for age group, sex, education level, marital status and employment sector

Box plots demonstrated the EQ-VAS distribution among the demographic variables. Figure 1 describes the box plots for EQ-VAS values within each category of the background variables.

Dissection of EQ-5D dimensions within categories of the demographic variables shows that patients up to 29 years of age had the lowest utility score, 0.50 (± 0.30) (Table 3). On average, EQ-VAS for women is 58.21 (± 17.62) and for men is 61.11 (± 16.44). WAD patients with a university education have the highest EQ-VAS average of 60.42 (± 17.73). However, the patients with other education have a low EQ-VAS average of 48.11 (± 11.591). Employment sectors do not show any significant differences in the mean value of EQ-VAS.

**Table 3:** Quality of life (EQ-5D) dimensions, EQ-VAS and WAD victims’ demographic factors

	Mobility		Self-care		Usual activity			Pain discomfort			Anxiety depression			EQ-VAS	EQ-5D
	No problem	Moderate problem	Moderate problem	Severe problem	No problem	Moderate problem	Severe problem	No problem	Moderate problem	Severe problem	No problem	Moderate problem	Severe problem	58.79 ±17.41	0.60 ± 0.25
**Age**															
Up to 29 *N* = 31	25 (80.6%)	6 (19.4%)	29 (93.5%)	2 (6.5%)	9 (29%)	21 (67.7%)	1 (3.3%)	0	20 (64.5%)	11 (35.5%)	9(29%)	22 (71%)	0	58.65 ± 18.74	0.50 ± 0.30
30 −49yrs *N* = 74	65 (87.8%)	9 (12.3%)	69 (93.2%)	5(6.8%)	31 (41.9%)	38 (51.4%)	5 (6.8%)	1 (1.4%)	63 (85.1%)	10 (13.5%)	27 (36.5%)	45 (60.8%)	2 (2.7%)	59.35 ±16.94	0.62 ±0.24
50 and above *N* = 32	29 (90.6%)	3 (9.4%)	31 (96.9%)	1 (3.1%)	16 (50%)	14 (43.8%)	2 (6.3%)	1 (3.1%)	27 (84.4%)	4 (22.5%)	11 (34.4%)	20 (62.5%)	1 (3.1%)	58.44 ±17.69	0.63 ±0.22
**Gender**															
Female *N* = 108	96 (88.9%)	12 (11.1%)	101 (93.5%)	7 (6.5%)	45 (32.8)	58 (53.7%)	5 (4.6%)	2 (1.9%)	85 (78.7%)	21 (19.4%)	34 (31.5%)	72 (66.7%)	2 (1.9%)	58.21 ± 17.62	0.59 ±0.26
Male *N* = 29	23 (79.3%)	6 (20.7%)	28 (96.6%)	1 (3.4%)	11(37.9%)	15 (51.7%)	3 (10.3%)	0	25 (86.2%)	4 (13.8%)	13 (44.8%)	15 (51.7%)	1 (3.4%)	61.11 ± 16.44	0.61 ±0.24
**Education**															
High school *N* = 68	60 (88.2%)	8 (11.8%)	63 (92.6%)	5 (7.4%)	23 (33.8%)	39 (57.4%)	6 (8.8%)	0	52 (76.5%)	16 (23.5%)	23 (33.8%)	44 (64.7%)	1 (1.5%)	58.76 ±17.44	0.55 ±0.27
University *N* = 60	51 (85%)	9 (15%)	57 (95%)	3 (5%)	30 (50%)	28 (46.7%)	2 (3.3%)	2 (3.3%)	51 (85%)	7 (11.7%)	23 (38.3%)	36 (60%)	1 (1.7%)	60.42 ±17.73	0.65 ±0.22
Others *N* = 9	6 (88.9%)	1(11.1%)	9 (100%)	0	3(33.3%)	6 (66.7%)	0	0	7 (77.8%)	1 (11.1%)	7 (77.8%)	1 (11.1%)	1 (0.7%)	48.11 ± 11.59	0.68 ± 0.28
**Marital status**															
Single *N* = 27	23 (85.2%)	4 (14.8%)	25 (92.6%)	2 (7.4%)	11 (40.7%)	16 (59.3%)	0	1 (3.7%)	22 (81.5%)	4 (14.8%)	8 (29.6%)	19 (70.4%)	0	59.96 ±20.16	0.63 ±0.24
Married/ live with partner *N* = 102	90 (88.2%)	12 (11.8%)	96 (94.1%)	6 (5.9%)	42 (41.2%)	52 (51%)	8 (7.8%)	1 (1%)	83 (81.4%)	18 (17.6%)	39 (38.2%)	61 (59.8%)	2 (2%)	58.78 ±16.53	0.60 ±0.25
Other *N* = 8	6 (75%)	2 (25%)	8 (100%)	0	3 (37.5%)	5 (62.5%)	0	0	5 (62.5%)	3 (37.5%)	0	7 (87.5%)	1(12.5%)	54.88 ±20.29	0.46 ±0.34
**Employment sector**															
White colour job *N* = 75	66 (88%)	9 (12%)	71 (94.7%)	4 (5.3%)	33 (44%)	39 (52%)	3 (4%)	2 (2.7%)	61 (81.3%)	12 (16.0%)	30 (40%)	43 (57.3%)	2 (2.7%)	59.45 ± 18.84	0.62 ±0.25
Blue colour job *N* = 48	40 (83.3%)	8 (16.7%)	44 (91.7%)	4 (8.3%)	19 (39.6%)	25 (52.1%)	4 (8.3%)	0	40 (83.3%)	8 (16.7%)	15 (31.3%)	32 (66.7%)	1 (2.1%)	57.75 ±14.79	0.58 ±0.25
Student or unemployed *N* = 14	13 (92.9%)	1 (7.1%)	14 (100%)	0	4 (28.6%)	9 (64.3%)	1 (7.1%)	0	9 (64.3%)	5 (35.7%)	2 (14.3%)	12 (85.7%)	0	58.79 ±18.73	0.51 ± 0.28
Consumes Medicine															
Yes *N* = 113	95 (84.1%)	18 (15.9%)	105 (92.9%)	8 (7.1%)	44 (38.9%)	62 (54.9%)	7 (6.2%)	2 (1.8%)	87 (77%)	24 (21.2%)	39 (34.5%)	72 (63.7%)	2 (1.8%)	58.04 ± 17.98	0.58 ±0.26
No *N* = 23	23 (100%)	0	23 (100%)	0	11 (47.8%)	11 (47.8%)	1 (4.3%)	0	22 (95.7%)	1 (4.3%)	7 (30.4%)	15 (65.2%)	1 (4.3%)	62.17 ±14.54	0.68 ±0.17
Lost income for WAD															
Yes *N* = 72	56 (77.8%)	16 (22.2%)	65 (90.3%)	7 (9,7%)	11 (15.3%)	53 (73.6%)	8 (11.1%)	0	52 (72.2%)	20 (27.8%)	15 (20.8%)	54 (75%)	3 (4.2%)	53.47 ± 16.26	0.50 ± 0.28
No *N* = 64	62 (96.9%)	2 (3.1%)	63 (98.4%)	1 (1.6%)	44 (68.8%)	20 (31.3%)	0	2 (3.1%)	57 (89.1%)	5 (7.8%)	31 (48.4%)	33 (51.6%)	0	64.44 ±16.86	0.70 ±0.17

For mobility and self-care the option ‘Severe problem’ has zero values and we did not put them in columns

Mobility and self-care have shown that irrespective of demographic background, participants have responded with mostly no problems, or very few have reported moderate problems. However, a higher proportion of men have moderate problems with their mobility. Higher proportions of women have severe problems with pain/ discomfort and moderate problems with anxiety and depression.

WAD patients who live alone have higher proportions of moderate problems. towards their usual activities. Higher proportions of married patients suffer severely from pain/discomfort while performing their daily activities. The societal values of EQ-5D-3 L showed the same trend as EQ-VAS for respective demographic variable categories.

**Fig. 2:**
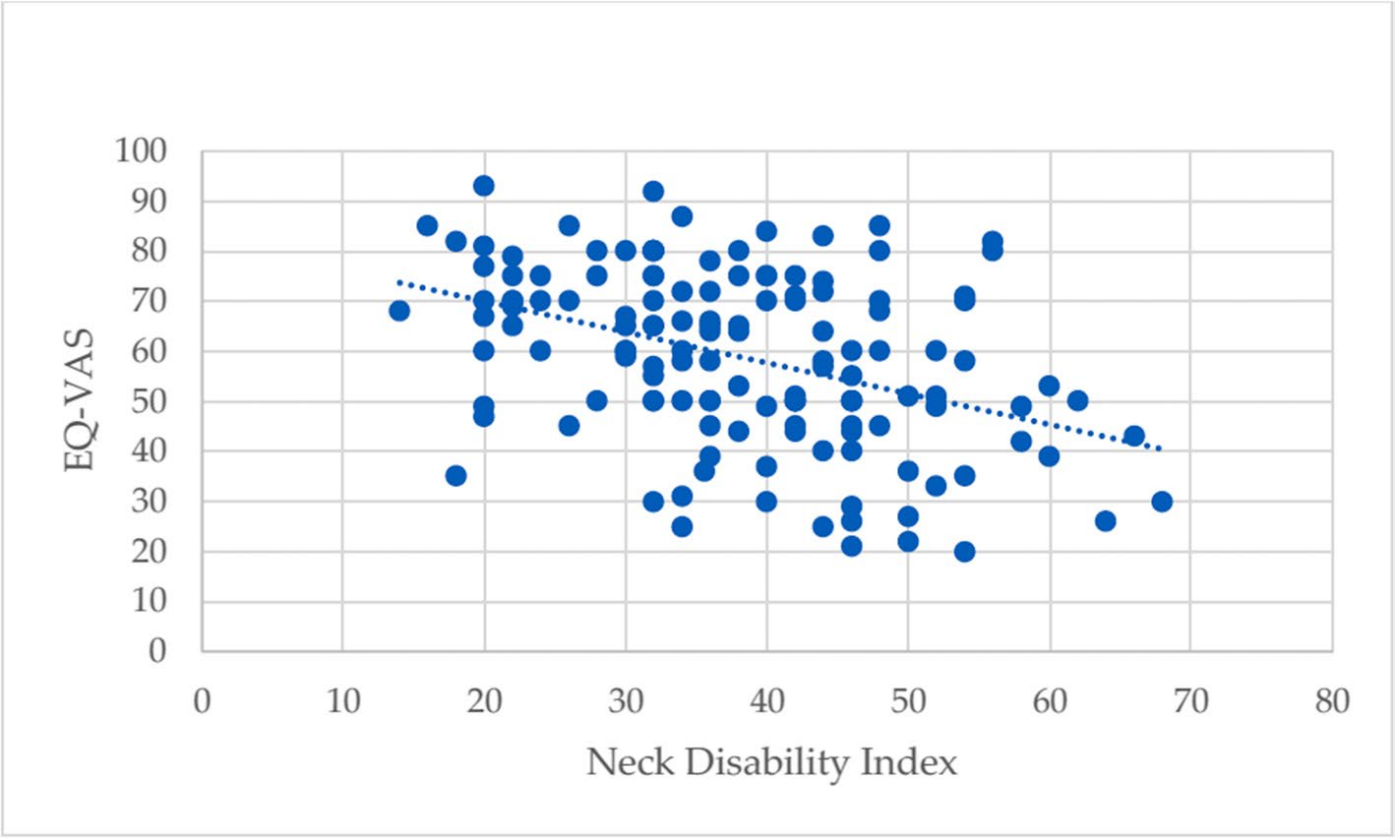
Distribution and regression line of EQ-VAS for NDI (independent variable)

A linear regression line was drawn for EQ-VAS where NDI is the predictor (Fig. 2) where, β is 82.346 (*p* < 0.001) and the NDI coefficient is −0.616 (level of significance *p* < 0.001).

## Discussions

The current study was the first to assess the quality of life (EQ-VAS) in chronic WAD patients. The majority of the WAD patients in the study are between 30 – and 49 years, women (93%), and high school educated. The mean EQ-VAS for the WAD patients in Sweden is 58.79 which is lower than EQ-VAS mean of patients from knee intervention (64.6), cruciate ligament intervention (62.8), Osteoarthritis (68.3) and higher than patients from lumbar spine intervention (47.8), hip intervention (56.3) and ankle intervention (55.8) in Swedish context [34]. The EQ-VAS mean score for Swedish general population is 79.5 [1]. Therefore, the policymakers should consider WAD patients with due emphasis as their overall HRQOL is relatively low.

Study participants who are married or living with a partner have the highest proportion of pain medicine consumption and their lost income from WAD. Probably, persons living with others in the same house need to perform household work, or could have child/ren, which might intensify their health problems, resulting in them consuming a higher proportion of pain medicine and more sick leaves, causing more income loss. However, these are suggestive explanations from Swedish social contexts. Further studies are warranted to better understand the causes of such medicine consumption and income loss. work absenteeism. Further studies are warranted to know the actual reasons.

The income loss due to WAD has the opposite proportional direction with age, which could be due to the fact that younger employees need more rigorous work-life balance [27], and could have a child at home, which could affect their WAD and hence HRQOL. The current study shows that men (OR 0.332, CI 0.111–0.994) are less likely to consume pain medicine than women due to WAD. Further qualitative studies are needed to understand if this is due to economic discrimination in the family, economic constraints, or other reasons. The current study has indicated that WAD men have used less WAD medicines, while in general Swedish women are more likely to access healthcare [29]. Tenenbaum et al. [33] reported that Swedish women commonly sought care later than men and that they more often sought primary health care at the first instance of care, while men more often sought hospital care as their first instance, this even though women had at least equal symptoms than men and need longer hospital stay. Therefore, further study is warranted for WAD women patients in Sweden for their health behaviour.

The patients with lower NDI scores had an obviously better HRQOL on the EQ-VAS. A lower NDI score means no or less neck disability, and a patient with no or less neck disability should have better HRQOL when the patients report their own utility through EQ-VAS. This is also supported by the existing literature [36]. One reason for that may be that women had half the neck muscle strength than men [20]. In the long-term outcome of anterior cervical decompression and fusion surgery for cervical disc disease male sex has been shown to be a predictor for improvement in neck-specific disability. While women reported more pain, disability and a worse psychosocial status, where psychosocial status and disability were related [12]. Also, in the present study, women have lower VAS and higher disability than men, which may be another reason for lower HRQOL. One may also speculate if the women both have paid work and, at the same time, household work is the main household responsibility.

The previous study [39] supports that university-educated WAD patients have better EQ-VAS scores than the lower-educated participants. Blue-collar employees with WAD had the lowest HRQOL in the current study. Blue-collar employees require more and/or exhaustive laborious job performance, which might cause further discomfort and pain to reduce their HRQOL.

Patients from the UK reported an average EQ-5D utility score of 0.59 three weeks after a whiplash injury [23]. At 12 months follow up, patients with mild (10–28% NDI) and moderate (30–48% NDI) disability were improved to 0.84 and 0.69 respectively but those with severe (50–68% NDI) disability reported slightly lower EQ-5D utility score 0.53 compared to baseline. In the present study, patients with moderate to severe disability 6 months to 5 years after the injury reported a low average utility score (0.60), and there is an urgent need to further develop effective rehabilitation strategies. This is especially true for those with severe (WAD grade III) as they are often excluded from intervention studies.

The study demonstrated that WAD patients who consume pain medicine have lower HRQOL (EQ-VAS: 58.04±17.98; EQ-5D-3 L: 0.58±0.26) than their peers who did not consume pain medicine (EQ-VAS: 62.17±14.54; EQ-5D-3 L: 0.68±0.17). The WAD patients who have lost their income due to sick leave have a major loss of their HRQOL (EQ-VAS: 53.47 ± 16.26; EQ-5D-3 L: 0.50 ± 0.28 ), while those who have not lost their income due to WAD have a better HRQOL (EQ-VAS: 64.44 ± 16.86; EQ-5D-3 L: 0.70±0.17). Swedish Social Insurance System (known as Forsäkringskassan in Sweden) can use the current findings noting that WAD patients have a higher proportion of moderate problems with their daily activities, moderate pain and discomfort and moderate level of anxiety and depression. It is noteworthy to mention here that majority of the WAD patients have no problems with their mobility and self-care. Therefore, the current study has significant policy implications in the Swedish context.

Understanding HRQOL is important to knowing the consequences of WAD, which can help with better decision-making across various demographic factors and assign necessary treatment priorities [11]. The current study has clearly demonstrated the patient-reported outcome measures of WAD patients in Sweden through EQ-5D questionnaires and EQ-VAS scores. The study limitation should mention the lower number (*n* = 137) of WAD patients. However, considering the lower prevalence of WAD in Sweden, the study has its own merits, especially the self-rated health status using EQ-VAS. This is the first HRQOL study among WAD patients in Sweden. EQ-VAS scores and EQ-5D-3 L societal values have indicated significant differences in the PROM of the WAD patients according to their sociodemographic factors. The findings of this study may have important contributions for the policymakers and well as to the WAD research community.

## Data Availability

The data is not publicly available for ethical reasons. However, interested researchers can contact the corresponding author to access the data for valid reasons.
